# Improving immune function and mitochondrial health in patients undergoing hemodialysis: benefits of combining high-flux dialysis with hemoperfusion

**DOI:** 10.3389/fmed.2026.1765737

**Published:** 2026-05-21

**Authors:** Qi Zhong, Qiudi Tu, Baoqiao Wu, Yuemin Liu, Yiwen Li, Bo Lin, Bin Zhu, Yan Ren

**Affiliations:** 1 Urology & Nephrology Center, Department of Nephrology, Zhejiang Provincial People’s Hospital (Affiliated People’s Hospital), Hangzhou Medical College, Hangzhou, Zhejiang, China; 2 Nephrology Center, The Fourth Affiliated Hospital Zhejiang University School of Medicine, Yiwu, Zhejiang, China

**Keywords:** hemoperfusion, high-flux dialysis, mitochondria, protein-bound toxins, T lymphocyte subsets

## Abstract

**Objective:**

This study aimed to investigate the effects of combining hemoperfusion (HP) with high-flux hemodialysis (HFD) on T lymphocyte subsets, mitochondrial health, and clinical outcomes in patients undergoing maintenance hemodialysis (MHD).

**Methods:**

In this single-center, prospective, self-controlled trial, 20 MHD patients received their regular HFD treatment, with the addition of a single HP session every 2 weeks for 12 sessions. Levels of protein-bound uremic toxins (PBUTs: indoxyl sulphate [IS] and p-cresyl sulphate [PCS]), T lymphocyte subsets (CD3+, CD4+, CD8+), mitochondrial membrane potential (MMP) in T cells, and patient-reported outcomes (SF-36, PSQI, pruritus scale) were assessed before and after the 12 sessions intervention.

**Results:**

Following a single HP + HFD session, IS and PCS levels decreased significantly (*p* < 0.001). However, their pre-perfusion levels at end were not significantly different from baseline. After 12 sessions, increases were observed in the levels of CD3+, CD4+, CD8 + cells, the CD4+/CD8 + ratio, and the percentages of CD3 + MMP, CD4 + MMP, and CD8 + MMP (all *p* < 0.05). Patients also demonstrated significant improvements in sleep quality (PSQI), uremic pruritus, and overall quality of life (SF-36) (all *p* < 0.01). Additionally, serum phosphorus and parathyroid hormone levels were significantly reduced after the treatment period.

**Conclusion:**

After 12 sessions of combined hemoperfusion and high-flux dialysis, we observed significant increases in T lymphocyte subsets (CD3+, CD4+, CD8+, CD4+/CD8 + ratio) and the percentage of T cells with preserved mitochondrial membrane potential. Patients also reported improved sleep quality, reduced pruritus, and better quality of life. However, pre-dialysis levels of protein-bound uremic toxins (indoxyl sulphate and p-cresyl sulphate) of last session were not significantly different from baseline, despite effective clearance immediately after each combined session. Therefore, the observed improvements in immune-related parameters cannot be mechanistically attributed to a sustained reduction in these toxins. Alternative explanations—such as intermittent attenuation of peak toxin concentrations, clearance of unmeasured solutes, or non-specific effects—remain possible. Given the single-arm, observational design, these findings are hypothesis-generating and require confirmation in randomized controlled trials.

## Introduction

The incidence of chronic kidney disease (CKD) in China has reached 8.2% (1). When CKD progresses to end-stage renal disease (ESRD), hemodialysis (HD), peritoneal dialysis (PD), or kidney transplantation are required to maintain life (2, 3). Hemodialysis is the most important treatment mode, chosen by almost 85% of patients (4). According to Yang et al. (2), the number of dialysis patients in China is expected to double from 2013 to 2025, with HD patients accounting for 92.12% of all patients.

Dialysis-associated complications, which range from severe disability to death, impose a huge medical burden. Adequate toxin clearance reduces complications in dialysis patients. Uremic toxins are usually classified into three categories (5): (1) Small molecule toxins, with a relative molecular weight < 500 Da, which are easily cleared by dialysis; (2) Medium-to-large molecular toxins, with a relative molecular weight > 500 Da, which can only be cleared by increasing transport capacity and dialysis membrane pore size (high-flux dialysis membrane); and (3) Protein-binding uremic toxins (PBUTs), with a high protein-binding rate that cannot be effectively cleared by regular hemodialysis, making them prone to accumulation.

In the past decades, clinical and experimental data have linked the accumulation of PBUTs, especially indoxyl sulphate (IS) and *p*-cresyl sulphate (PCS), with the pathophysiology of CKD-related complications (6, 7), such as vascular calcification, atherosclerosis (8–10), anemia (11), insulin resistance (12), and bone mineral diseases (13). Increasing evidence supports the involvement of IS and PCS in CKD-related immune dysfunction. Excessive accumulation of PBUTs can activate innate immunity and alter adaptive immune responses. By damaging anti-inflammatory presenting cells, cytotoxic T cells and helper T lymphocytes undergo premature aging and cellular immune deficiency (14). The repeated hemodialysis process significantly disrupts the balance between activated T cells and regulatory T cells (Tregs) in patients with ESRD. Normal responses of immune cells can be reduced, leading to infectious diseases. T cells are affected by uremic toxins (15).

Hemoperfusion—a special blood purification mode—effectively removes PBUTs through adsorption (16). Thus, we aimed to investigate whether a combination of high-flux hemodialysis and hemoperfusion can improve mitochondrial and T lymphocyte function by increased PBUTs clearance.

## Materials and methods

This trial was approved by the ethical review board of Zhejiang Provincial People’s Hospital (approval number 2021KY055).

### Patients

This single-center prospective study included 20 patients who received maintenance hemodialysis (MHD) treatment in our hospital between October 2021 and January 2023. The primary disease included various types of glomerulonephritis, diabetic nephropathy, and secondary systemic disease. All enrolled patients provided signed informed consent.

The inclusion criteria were as follows: male or female aged ≥ 18 years old; MHD > 6 months, regular dialysis 3 times/week, 4 h/time; the vascular pathway is an internal fistula; and voluntary testing.

The exclusion criteria were as follows: coagulation disorders, severe bleeding tendencies, and active bleeding; serum albumin < 30 g/L; Kt/V < 1.2; low blood pressure; severe cardiopulmonary dysfunction; acute infections; severe heart, lung, liver, and nervous system diseases; other critical conditions; and malignant tumors.

### Sample size

This was a prospective, self-controlled, paired design, longitudinal trial. The PBUT level of patients before and after blood perfusion was the observation indicator. According to the pre-experiment results (Supplementary Figure S1), the average concentration of IS before hemoperfusion was 31.71 μg/mL, and that after hemoperfusion was 25.11 μg/mL, with an estimated standard deviation of 5.61 μg/mL. With a two-sided *α*-level of 0.05 and 90% confidence level, a required sample size of 17 cases was estimated using PASS 15 software. Accounting for 20% of cases being lost to follow-up, at least 20 patients were recruited.

### Treatment regimens

All selected patients were given phosphorus binders for phosphorus reduction, folic acid, iron, and erythropoietin or roxadustat for anemia correction. Patients with hypertension were given antihypertensive drugs to control their blood pressure level, and patients with diabetes were treated with insulin or hypoglycaemic drugs to control their blood glucose levels.

The 20 patients who had previously undergone high-flux hemodialysis were selected for the self-controlled study, and high-flux hemodialysis combined with hemoperfusion was performed. Treatments were based on thrice-weekly dialysis sessions, 4 h per session, with a blood flow of 220–280 mL/min and a dialysate flow of 500 mL/min. For dialysis filter, disposable high-flux polysulfone membrane (Revacelear 300, polyarylethersulfone membrane, Jinbao Kidney Care Products [Shanghai] Co., Ltd.) was used in hemodialysis. The dialyser has a membrane area of 1.4 m^2^, an ultrafiltration coefficient of 35 mL/h/mmHg, and a reported *β*₂-microglobulin clearance of >20 mL/min. Non-reusable perfusion (KHA80, 130 g, Zhuhai Jianfan Biotechnology Co., Lt) was selected for hemoperfusion which contains 130 g of styrene-divinylbenzene copolymer resin as the adsorbent material, with a specific surface area of approximately 1,000–1,200 m^2^/g. The adsorption capacity for protein-bound uremic toxins, including indoxyl sulphate and p-cresyl sulphate, has been reported by the manufacturer to be >200 μg/g of adsorbent under *in vitro* conditions. We used the KHA80 disposable resin perfumer and connected it in series before the dialyser for 2 h of continuous perfusion. After reaching saturation, the perfumer was removed, and high-flux hemodialysis was continued for another 2 h. We performed hemodialysis thrice weekly and hemodialysis + hemoperfusion once every two weeks; follow-up observation was conducted for 24 weeks.

### Clinical assessment

Blood samples before and after the first perfusion and before the last perfusion were collected. We used liquid chromatography–tandem mass spectrometry to detect differences in the content of PCS and IS (Supplementary material 1). Flow cytometry was used to detect the status of various subsets of T lymphocytes, combined with laboratory indicators, such as hemoglobin, phosphorus, calcium, and parathyroid hormone (PTH), alongside 36-Item Short Form Health Survey (SF-36) and Pittsburgh Sleep Quality Index (PSQI) scores to evaluate improvements in quality of life. The SF-36 scale provides an objective measurement of nutritional status, which can indicate overall health status, and is widely used in clinical research. The scoring method of the PSQI sleep scale involves multiple aspects, including self-evaluation of sleep quality, time to fall asleep, sleep time, sleep efficiency, sleep disorders, and other dimensions of consideration. Through these comprehensive evaluations, an individual’s sleep quality can be comprehensively and accurately evaluated. Considering itching is a common symptom in dialysis patients, we applied the modified Duo’s itching score, which is commonly used to evaluate the degree of itching.

### Mitochondrial membrane potential (MMP) assay

MMP measurements were outsourced to a commercial laboratory. According to the laboratory‘s standard protocol, peripheral blood mononuclear cells were isolated by Ficoll-Paque density gradient centrifugation. MMP was assessed using JC-1 (Thermo Fisher, T3168) at 2 μg/mL. Cells were stained with JC-1 for 30 min at 37 °C, then labeled with anti-CD3-FITC, anti-CD4-PE, and anti-CD8-APC antibodies. Propidium iodide (1 μg/mL) was used to exclude dead cells. A positive control for depolarisation (50 μM CCCP, 15 min) was included in each batch. Data were acquired on a BD FACSCanto II flow cytometer and analyzed with FlowJo v10.8. However, the raw data files and detailed gating strategy were not provided to us by the outsourcing laboratory; therefore, the gating strategy could not be shown. MMP was reported as the percentage of CD3+, CD4+, or CD8 + cells with preserved membrane potential (high JC-1 red/green ratio). Readers should interpret these results with caution given the limited methodological detail available.

## Statistical analysis

Statistical analyses were performed using SPSS version 27.0 (IBM Corp., Armonk, NY, USA). Normality of continuous variables was assessed using the Shapiro–Wilk test. Normally distributed data are presented as mean ± standard deviation (SD), and non-normally distributed data as median with interquartile range (IQR).

For comparisons between baseline and 12 sessions follow-up (paired measurements), paired *t*-tests were used for normally distributed variables, and Wilcoxon signed-rank tests for non-normally distributed variables.

Given the exploratory, hypothesis-generating nature of this single-arm pilot study, no adjustment for multiple comparisons was applied. A total of approximately 15 outcome variables were tested. To reduce the risk of type I error, all findings should be interpreted as descriptive associations requiring confirmation in independent studies. *p*-values are reported as nominal values without correction; they are not intended for confirmatory inference.

## Results

### Study participants and follow-up

Overall, 20 patients undergoing hemodialysis were enrolled and followed up for 12 sessions. Due to the impact of the coronavirus disease 2019 pandemic, the hemoperfusion time was slightly adjusted; nonetheless, it was typically conducted once every 2 weeks. All enrolled patients completed the study.

### Baseline characteristics

The 20 participants had a mean age of 53.1 ± 8.7 years and had undergone dialysis for 5.0 (range, 3.0–7.0) years; 70% were male. The access was all arteriovenous fistulas. Primary diseases included chronic nephritis (60%), diabetes nephropathy (25%), hypertensive nephropathy (5%), obstructive nephropathy (5%), and polycystic kidney (5%).

### Primary outcomes

#### PBUT concentrations

The concentration of PBUTs significantly reduced from before to after a single perfusion (IS: 11.56 ± 5.65 vs. 7.34 ± 3.91 μg/mL, *p* < 0.001; PCS: 21.29 (4.50–31.90) vs. 16.16 (3.07–26.15) μg/mL, *p* < 0.001), indicating that high-flux dialysis combined with hemoperfusion can clear PBUTs. At the end, 12 high-flux dialysis combined with perfusion tests were conducted. Blood samples taken before the last perfusion revealed PBUT levels were not significantly decreased (IS: 11.56 ± 5.65 vs. 13.82 ± 10.87 μg/mL, *p* = 0.267; PCS: 21.29 (4.50–31.90) vs. 19.84 (9.02–28.53) μg/mL, *p* = 0.502). As shown in Table 1.

**Table 1:** Differences in PBUTs and lymphocyte subsets before and after a single treatment and before the last treatment.

Variable	Before first perfusion (*n* = 20)	After first perfusion (*n* = 20)	Before last perfusion (*n* = 20)	*P*1 -value (single session)	*P*2 -value (long‑term)
IS (μg/mL)	11.56 ± 5.65	7.34 ± 3.91	13.82 ± 10.87	<0.001	0.267
PCS (μg/mL)	21.29 (4.50, 31.90)	16.16 (3.07, 26.15)	19.84 (9.02, 28.53)	<0.001	0.502
CD3^+^ (%)	0.65 ± 0.08	0.67 ± 0.09	0.70 ± 0.09	<0.01	
CD4^+^ (%)	0.39 ± 0.10	0.40 ± 0.08	0.44 ± 0.08	<0.01	
CD8^+^ (%)	0.23 ± 0.08	0.24 ± 0.07	0.25 ± 0.06	0.015	
CD4^+^/CD8^+^	1.49 (1.07, 2.35)	1.78 (1.16, 2.64)	1.93 (1.38, 2.99)	<0.01	
Absolute counts of CD3^+^	579.32 ± 171.44	491.09 ± 224.53	572.17 ± 267.44	0.17	
Absolute counts of CD4^+^	362.53 (233.97, 3 82.15)	241.19 (182.89, 362.06)	336.23 (237.78, 430.89)	0.091	
Absolute counts of CD8^+^	181.85 (138.96, 243.61)	142.55 (89.37, 235.33)	166.96 (88.88, 279.53)	0.022	
CD3^+^ MMP (%)	0.38 ± 0.21	0.31 ± 0.19	0.53 ± 0.18	<0.01	
CD4^+^MMP (%)	0.25 (0.15, 0.35)	0.21 (0.13, 0.30)	0.48 (0.25, 0.61)	<0.01	
CD8^+^MMP (%)	0.47 ± 0.2	0.40 ± 0.21	0.62 ± 0.16	<0.01	

PBUTs, protein-bound toxins; IS, indoxyl sulphate; PCS, p-cresyl sulfate; MMP, Mitochondrial membrane potential.

P1, comparison between pre and after a single session; P2, comparison between pre and pre-last.

#### Changes in T lymphocyte subsets

The levels of CD3 + (0.65 ± 0.08% vs. 0.70 ± 0.09%), CD4 + (0.39 ± 0.10%vs. 0.44 ± 0.08%), CD8 + (0.23 ± 0.08% vs. 0.25 ± 0.06%) and the CD4+/CD8 + ratio [1.49 (1.07, 2.35) vs. 1.93 (1.38, 2.99)] showed an increase from baseline (nominal *p* < 0.05). There was no statistically significant difference between the absolute counts of CD3 + and CD4 + before and after treatment (*p* > 0.05), while the absolute counts of CD8 + [181.85 (138.96, 243.61) vs. 166.96 (88.88, 279.53)] decreased (*p* < 0.05). Given the small effect size (absolute difference 0.05%) and overlapping standard deviations, the clinical significance of this change is uncertain, and it may not remain significant after correction for multiple comparisons. As shown in Table 1.

#### Changes in mitochondrial levels

The percentages of CD3 + MMP (0.38 ± 0.21% vs. 0.53 ± 0.18%), CD4 + MMP (0.25 (0.15, 0.35)% vs.0.48 (0.25, 0.61)%), and CD8 + MMP (0.47 ± 0.2%vs.0.62 ± 0.16%) showed an increase from baseline (*p* < 0.05). As shown in Table 1.

#### Changes in patient scores before and after treatment

The PSQI (8.95 ± 3.07 vs. 7.05 ± 2.46, *p* < 0.01), itching (modified Duo’s VAG Scale: 4 (0–11.75) vs. 0 (0–7.5) *p* < 0.01), and quality of life (SF-36: 78.65 ± 11.77 vs. 82.75 ± 8.96 *p* < 0.01) scores showed significant improvements from baseline. As shown in Table 2.

**Table 2:** Changes in the scale scores before and after the treatment.

Variable	Before first perfusion (*n* = 20)	After first perfusion (*n* = 20)	Before last perfusion (*n* = 20)	*p*
PSQI	8.95 ± 3.07	8.10 ± 2.43	7.05 ± 2.46	<0.01
SF-36	78.65 ± 11.77	81.10 ± 10.45	82.75 ± 8.96	<0.01
Dou’s pruritus scale scores	4.00 (0, 11.75)	0 (0, 10.00)	0 (0, 7.50)	<0.01

PSQI, Pittsburgh sleep quality index; SF-36, 36-item Short-Form.

### Secondary outcomes

#### Changes in albumin and hemoglobin levels

No statistically significant change was observed of the albumin level (38.70 (36.18, 40.25) g/L vs.36.70 (35.20, 38.60) g/L) (*p* = 0.08), while there was no statistically significant change in the hemoglobin level (*p* > 0.05). As shown in Table 3.

**Table 3:** Changes in clinical laboratory indicators before and after the treatment.

Variable	Before first perfusion (*n* = 20)	Before last perfusion (*n* = 20)	*p*
Alb (g/L)	38.70 (36.18, 40.25)	36.70 (35.20, 38.60)	0.08
Hb (g/L)	107.45 ± 12.93	106.20 ± 13.03	0.14
Ca (mmol/L)	2.21 ± 0.14	2.26 ± 0.12	0.09
P (mmol/L)	2.06 (1.82, 2.41)	1.46 (1.23, 2.02)	0.02
PTH (pg/mL)	408.44 ± 261.16	249.55 ± 145.77	<0.01
β2-MG^pre^ (mg/L)	29.20 (24.95, 33.13)	30.6 (27.00, 32.85)	0.07
β2-MG^post^ (mg/L)	12.10 (9.63, 15.90)	12.35 (10.03, 17.40)	0.05

Alb, albumin; Hb, hemoglobin; Ca, calcium; P, Phosphorus; PTH, parathyroid hormone.

β2-MG^pre^, β2-microglobulin before the blood purification; β2-MG^post^, β2-microglobulin after the blood purification.

#### Changes in calcium, phosphorus, PTH, and β2-MG levels

There were no statistically significant differences in calcium (*p* = 0.09), pre-dialysis β2-microglobulin (*p* = 0.07), or post-dialysis β2-microglobulin (*p* = 0.05) from baseline to the end. Of course, there was a significant decrease in β2-microglobulin before and after single perfusion, which was statistically significant (*p* < 0.001). After 12 sessions of treatment, levels of phosphorus (2.06 (1.82, 2.41) mmol/Lvs.1.46 (1.23, 2.02) mmol/L) and PTH (408.44 ± 261.16 pg. /mL vs. 249.55 ± 145.77 pg./mL) were significantly lower than those before treatment (*p* = 0.022 and *p* < 0.01, respectively). However, because concomitant medications (phosphate binders, active vitamin D, etc.) were not quantitatively tracked, these changes cannot be attributed specifically to the HP + HFD intervention. As shown in Table 3.

## Discussion

PBUTs such as IS and PCS, derived from dietary amino acid metabolism, are difficult to remove by conventional dialysis and account for approximately 25% of known uremic toxins (17). IS and PCS constitute >90% of PBUTs and are associated with all-cause mortality in CKD (18). These toxins induce oxidative stress, worsen renal function, promote vascular calcification, and increase cardiovascular events; PCS is an independent risk factor for plaque formation (19). Previous studies have shown that activated carbon can enhance PBUT clearance (20), and *in vitro* experiments suggest that combining hemoperfusion with hemodialysis may improve toxin removal (21). In our study, a single HP + HFD session significantly reduced IS and PCS, but this reduction was not sustained in the end, possibly due to insufficient bi-weekly perfusion frequency. Increasing perfusion frequency or using a larger adsorber (e.g., KHA80) might enhance clearance and improve outcomes.

ESRD patients exhibit marked immune dysregulation involving uremic toxins, vitamin D metabolism, chronic inflammation, and dialysis itself (22). A complex interplay between innate and adaptive immunity, with coexisting activation and suppression, has been described (23). Lymphocyte decline predicts mortality in MHD patients (24). T lymphocyte subsets, particularly the CD4+/CD8 + ratio, are key immune indicators; a ratio <1 is linked to higher morbidity and mortality (25).

In this single-arm study, after 12 sessions of HP + HFD treatment, we observed significant increases in the levels of CD3+, CD4+, CD8+, the CD4+/CD8 + ratio, and the percentages of CD3 + MMP, CD4 + MMP, and CD8 + MMP. These temporal associations are consistent with a favorable shift in T cell profile and mitochondrial health, but causation cannot be inferred due to the lack of a control group. Beyond statistical significance, the clinical significance of the observed changes warrants emphasis. The improvement in CD4+/CD8 + ratio to 1.93 represents a restoration of immune homeostasis which if confirmed in future trials may reduce infection risk.

Sleep disorders are common in MHD patients, with a prevalence as high as 77.0% in older individuals (26). Poor sleep quality is an independent risk factor for cognitive impairment (26). In our study, PSQI scores improved significantly after HP + HFD (*p* < 0.01), which may be associated with the clearance of uremic toxins and subsequent reduction in inflammation and itching.

Uremic pruritus (UP) affects 51–81% of MHD patients (27). Its pathogenesis is multifactorial, involving secondary hyperparathyroidism, calcium-phosphorus imbalance, insufficient dialysis, local inflammatory mediators, and opioid receptor imbalances (28). UP negatively impacts quality of life, sleep, and mood, and is associated with increased all-cause, cardiovascular, and infection-related mortality (29). Our patients showed a significant decrease in the modified Duo Itch Scale score after treatment (*p* < 0.01), which was directionally consistent with marked improvement in itching.

Quality of life, as measured by SF-36, is generally lower in MHD patients than in the general population (30). Our baseline SF-36 score (78.65) was consistent with this, and after 12 sessions of HP + HFD, the SF-36 score showed a significant change from baseline (*p* < 0.01), with direction consistent with improved quality of life.

Chronic kidney disease patients often develop bone mineral disorders; long-dialysis patients frequently have calcium-phosphorus abnormalities and secondary hyper- parathyroidism, increasing the risk of renal bone disease and vascular calcification. Our patients had phosphorus levels exceeding KDIGO targets and were at high cardiovascular risk. Large studies (DOPPS, CORES, COSMOS) have shown that disturbances in calcium, phosphorus, and PTH increase all-cause and cardiovascular mortality (31–33). For example, Gutierrez et al. (34) reported a 20% higher mortality risk when blood phosphorus exceeded 5.5 mg/dL. Achieving all three guideline targets for Ca, P, and PTH can significantly improve survival; failure to meet any one increases mortality risk by 15–21%, and failure to meet all three by 51% (35). After 12 sessions of HP + HFD, our patients had reduced serum phosphorus and PTH levels. An important limitation is the lack of quantitative documentation of concomitant medication doses. While the protocol instructed that phosphate binders, erythropoiesis-stimulating agents, active vitamin D, antihypertensives, and glucose-lowering agents be maintained at stable doses, we did not systematically record dose adjustments over the 6-month follow-up. Therefore, the observed reductions in serum phosphorus and PTH, as well as other biochemical changes, may reflect routine medical optimization during protocolised follow-up rather than a direct effect of hemoperfusion. Consequently, we make no causal claim regarding the effect of HP + HFD on these biochemical parameters.

The near-complete resolution of pruritus (median score from 4 to 0) is particularly noteworthy, as uremic itching severely impairs quality of life and is notoriously difficult to treat. Furthermore, the reduction in serum phosphorus to within KDIGO target range and the substantial decrease in PTH levels have well-established benefits for bone health and cardiovascular outcomes. Collectively, these clinically meaningful improvements support the use of combined HP + HFD as a valuable adjunctive therapy in selected MHD patients.

An unexpected finding was the dissociation between PBUT clearance and clinical outcomes. Despite no sustained reduction in pre-dialysis IS/PCS in the end, we observed sustained improvements in immune parameters, mitochondrial health, sleep, pruritus, and quality of life. This dissociation directly refutes a simple causal model in which clinical benefits are mediated by persistently lower pre-dialysis PBUT concentrations. Several speculative mechanisms may explain this paradox: intermittent attenuation of peak toxin concentrations (“peak concentration hypothesis”), clearance of unmeasured uremic toxins or inflammatory mediators, self-sustaining mitochondrial recovery, or limitations of single pre-dialysis toxin sampling. However, these remain post-hoc hypotheses. Alternative explanations (placebo effect, regression to the mean, unmeasured dietary or medication changes) cannot be excluded. The lack of a control group precludes definitive attribution of benefits to HP. Therefore, these results should be considered preliminary and hypothesis-generating. Future randomized controlled trials are needed to establish causality.

This study has several limitations. First, its single-center design and small sample size (*n* = 20) limit the statistical power and generalizability of our findings. Second, the single-center, non-randomized design precludes definitive causal conclusions. However, this study was intended as a pilot investigation to generate hypotheses. The consistent improvements across multiple endpoints, coupled with the temporal sequence of changes, provide preliminary evidence that warrants confirmation in future randomized controlled trials. Third, the 6-month follow-up period was sufficient to demonstrate short-term improvements in immune parameters and quality of life but is too short to evaluate long-term outcomes. Fourth, this study did not include quantitative monitoring of dietary intake, which could influence toxin generation. However, all patients maintained a consistent, physician-prescribed renal diet throughout the study. Fifth, the absence of direct measurements of systemic inflammatory markers (e.g., interleukins) is a limitation. Future studies should incorporate these measures to more directly elucidate the anti-inflammatory mechanisms of combined hemoperfusion and high-flux dialysis. Sixth, concomitant medications were not quantitatively tracked. Although the study protocol required stable dosing of phosphate binders, ESA, iron, active vitamin D, antihypertensives, and hypoglycaemic agents, we did not collect dose records at each visit. Thus, we cannot exclude the possibility that changes in biochemical parameters (e.g., phosphorus, PTH) were influenced by unrecorded medication adjustments. This confounder further precludes any causal attribution of the observed biochemical changes to the HP + HFD intervention. Seventh, no adjustment was made for multiple comparisons. With approximately 15 outcome variables tested, the probability of at least one false-positive finding is substantially higher than 0.05. Therefore, all reported *p*-values should be interpreted as nominal, and the findings should be considered exploratory. Confirmatory studies with pre-specified primary endpoints and appropriate correction for multiplicity are needed. Eighth, the MMP assay was performed by an external laboratory, and we do not have access to the raw flow cytometry data or the complete gating strategy. While the laboratory confirmed the use of JC-1, CCCP control, and PI viability gating, the lack of a detailed gating figure precludes full methodological transparency and reproducibility. This limitation should be considered when interpreting the MMP findings.

Despite these limitations, the consistent improvements across multiple parameters provide a strong rationale for future large-scale, randomized, multicenter, long-term trials. Such studies should compare HP + HFD versus HFD alone, focus on hard clinical endpoints, and incorporate mechanistic investigations of inflammatory pathways and broader toxin clearance.

Therefore, while the results are promising, they should be interpreted as preliminary and hypothesis-generating. Future randomized controlled trials are needed to determine whether the clinical improvements are causally related to HP-mediated toxin clearance, and if so, through which mechanisms.

## Conclusion

In this single-arm pilot study, 12 sessions of combined hemoperfusion and high-flux dialysis was associated with favorable changes in T lymphocyte subsets and mitochondrial membrane potential in MHD patients. However, because pre-dialysis levels of indoxyl sulphate and p-cresyl sulphate did not decrease sustainably (*p* = 0.267 and *p* = 0.502), the mechanistic premise that sustained PBUT reduction drives these immune changes is not supported by our data. While single-session clearance was effective, the lack of long-term toxin reduction indicates that the observed immunological improvements must involve pathways other than steady-state PBUT lowering. These results should be considered exploratory. Future randomized controlled trials are needed to establish causality and elucidate mechanisms.

## Data Availability

The original contributions presented in the study are included in the article/Supplementary material, further inquiries can be directed to the corresponding author.
