# Supplementary material for: Improving immune function and mitochondrial health in patients undergoing hemodialysis: benefits of combining high-flux dialysis with hemoperfusion
Source: Front Med (Lausanne). 2026 May 21;13:1765737. doi: 10.3389/fmed.2026.1765737 (PMC13233219; doi:10.3389/fmed.2026.1765737)
Supplement: Supplementary file 1 [file Data_Sheet_1.doc]

**Supplementary Figure S1 Pre experimental results**


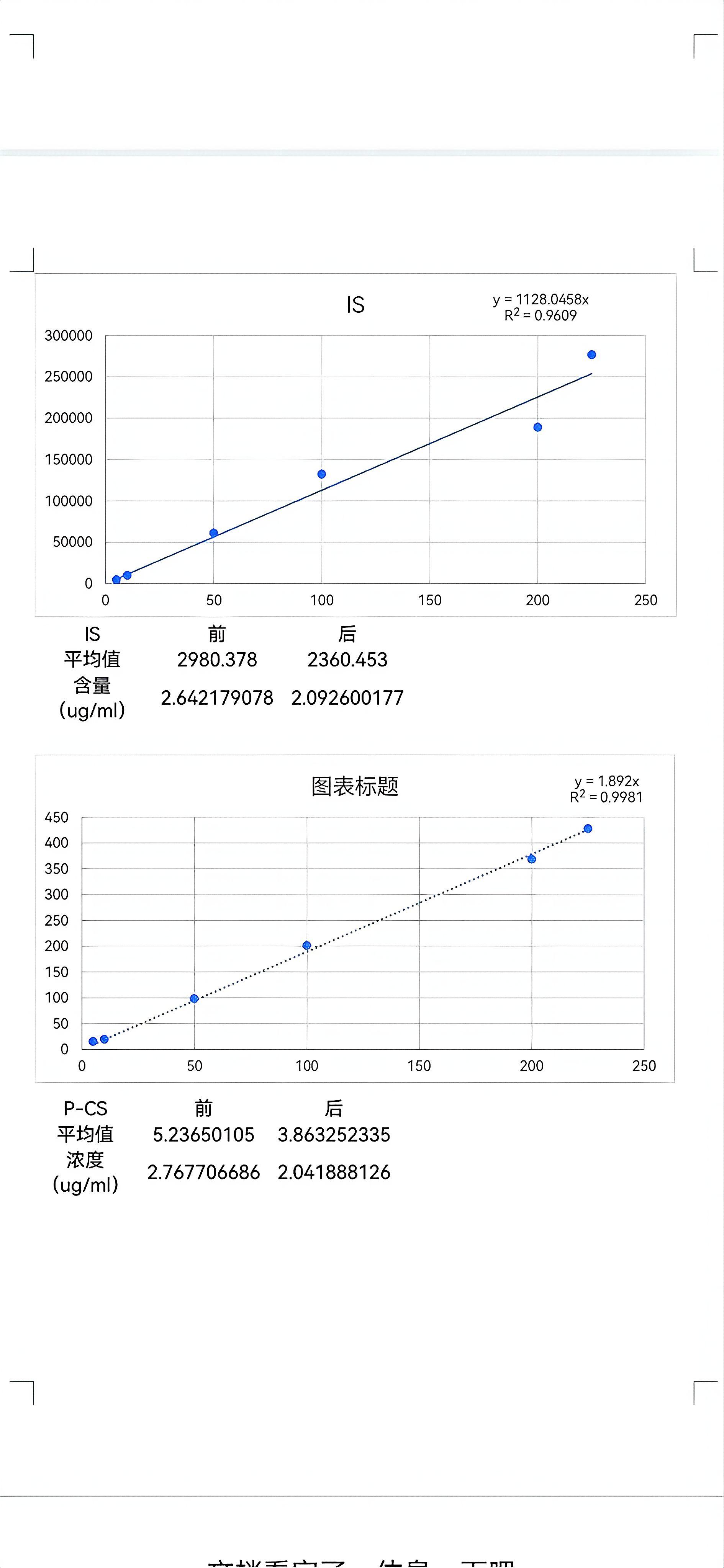


IS pre post

mean 2980.378 2360.453

content 31.71 25.11

(ug/mL)


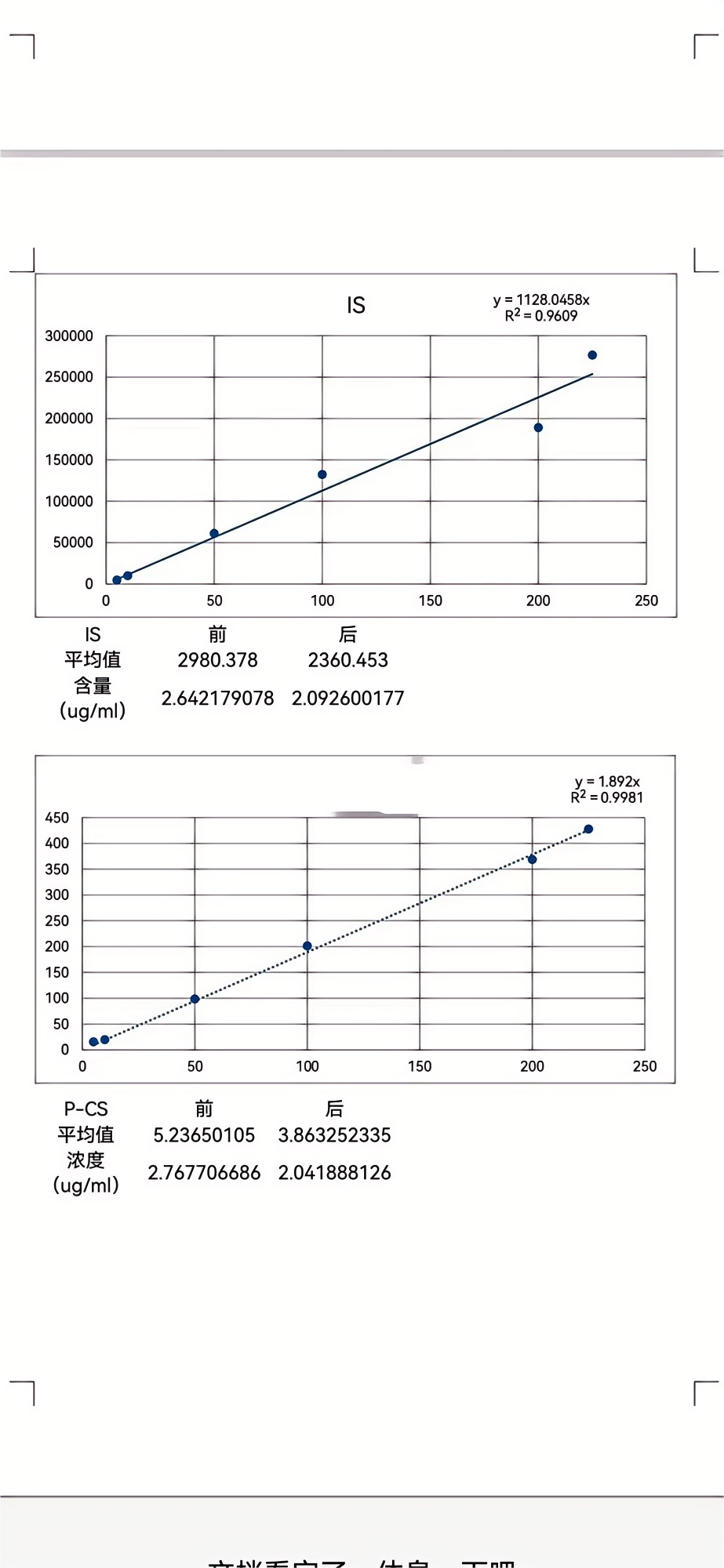


Pcs pre post

mean 5.2365105 3.863252335

content 2.767706686 2.041888126

(ug/mL)
