# Supplementary material for: Improving immune function and mitochondrial health in patients undergoing hemodialysis: benefits of combining high-flux dialysis with hemoperfusion
Source: Front Med (Lausanne). 2026 May 21;13:1765737. doi: 10.3389/fmed.2026.1765737 (PMC13233219; doi:10.3389/fmed.2026.1765737)
Supplement: Supplementary file 2 [file Supplementary_file_1.doc]

**Supplementary materials**

**Concentration determination of IS, PCS in blood**

1.Preparation of internal standard: weigh 1mg of chloramphenicol standard and hydrochlorothiazide to methanol and methanol in a 10 mL volumetric flask with 100 μg/mL of mother liquor A, B.

2.Preparation of standard solution of IS and PCS: weigh 5mg of IS and PCS potassium salt dissolved in methanol, A concentration of 200 μg/mL mother liquor C was obtained with methanol in a 25 mL volumetric flask, D, 0.01 mL of mother liquor A, respectively, B and 5mL, 2.5mL, 0.5mL, 0.25mL, 0.05mL, 0.025mL mother liquor C, D And diluted into 100 ng/mL, 50 μg/mL, 10 μg/mL, 10 μg/mL, 5 μg/mL, 1 μg/mL, 0.5 μg/mL standard, in a 10 mL volumetric flask.

3.Human blood (pre-dialysis and after dialysis) : Human blood samples were stored at-8℃. During pretreatment, 100 μ L samples were stored in 2 mL polypropylene tubes, adding 500 μ L, 100 ng/mL (last 12 microliters of internal standard parent solution) of methanol standard (hydrochlorothiazide and chloramphenicol) solution for 1min, centrifuged at 13000rpm for 100 μ L of supernatant and 100 μ L of purified water.

4. assaying

Conditions: HPLC condition: Venusil XBP Phenyl column (10032.1 mm, 5 m m); mobile phase ratio: mobile phase A: 2 mmol/L ammonium acetate dissolved in 0.1% formic acid, mobile phase B: methanol (A: B=50:50); flow rate of 0.3 mL/min; column temperature 40℃; autosampler temperature 4℃.

MS condition: API 3000 three-four pole instrument (Applied Biosystems, Toronto, on, Canada) for multiple reaction monitoring mode.

ESI condition: Turbine spray voltage is set to-4200 v; the source temperature is maintained at 500℃.

electron pair:

| chemical compound | Ion mode | Parent ion (m / z) | Subion (m / z) |
| --- | --- | --- | --- |
| IS | ESI - | 121.1 | 81.1 |
| Hydrochlorothiohydrogen | ESI - | 296.2 | 226.8 |
| PCS | ESI - | 187.0319 | 107.0461 |
| chloramphenicol | ESI - | 321.108 | 152.085 |
